# Non-thermal gliding arc plasma for cesium-137 mobilization and radiological risk reduction in electric arc furnace dust

**DOI:** 10.1039/d6ra06604f

**Published:** 2026-10-06

**Authors:** Vikas Rathore, Klitsadee Yubonmhat, Arlee Tamman, Dechanun Choomjun, Shaima Masae, Pattarachai Bor-in, Kewalee Nilgumhang, Grittima Kongprawes, Tejal Barkhade, Arunsinh B. Zala, Subash C. B. Gopinath, Tijjani Adam, Siti Khadijah Zaaba, Falguni Bhabhor, Sujoy Chattaraj, Mudtorlep Nisoa

**Affiliations:** a Agriculture and Bio Plasma Technology Center, Science and Technology Park, Chiang Mai University Chiang Mai 50100 Thailand; b Nuclear and Radiation Safety Research Section, Nuclear Safety Technology and Radioactive Waste Management Center, Thailand Institute of Nuclear Technology Nakhon Nayok 26120 Thailand; c Center of Engineering and Nuclear Technology, Thailand Institute of Nuclear Technology Nakhon Nayok 26120 Thailand; d Faculty of Thailand Academy of Sciences (TAS), Mahidol University Nakhon Pathom 73170 Thailand; e Department of Physics, Faculty of Science, King Mongkut's Institute of Technology Ladkrabang Bangkok 10520 Thailand; f Nuclear Engineering and Technology Center (NETC), Tokamak Section (TS), Thailand Institute of Nuclear Technology Nakhon Nayok 26120 Thailand; g Department of Life Sciences, Parul Institute of Applied Sciences, Parul University Vadodara-391760 Gujarat India; h Natural Sciences and Science Education, National Institute of Education, Nanyang Technological University Singapore 637616 Singapore; i Center for Global Health Research, Saveetha Medical College & Hospital, Saveetha Institute of Medical and Technical Sciences (SIMATS) Thandalam Chennai 602105 Tamil Nadu India; j Faculty of Chemical Engineering & Technology, and Institute of Nano Electronic Engineering, Universiti Malaysia Perlis (UniMAP) 02600 Arau Kangar 01000 Perlis Malaysia; k Department of Technical Sciences, Western Caspian University Baku AZ 1075 Azerbaijan; l Faculty of Electronics Engineering & Technology, Universiti Malaysia Perlis Arau 02600 Perlis Malaysia; m Bhavan's Seth R.A. College of Science, Gujarat University Ahmedabad 380001 India; n Department of Chemical Engineering, Thapar Institute of Engineering and Technology Patiala-147004 Punjab India; o Futuristic Science Research Center, School of Science, Walailak University Nakhon Si Thammarat 80160 Thailand nmudtorl@wu.ac.th; p Functional Materials and Nanotechnology Center of Excellence, School of Science, Walailak University Nakhon Si Thammarat 80160 Thailand

## Abstract

Electric arc furnace dust (EAFD) contains hazardous metals and radionuclides such as ^137^Cs^+^ immobilized within Fe- and Zn-based oxide matrices, posing environmental and radiological risks. This study investigates non-thermal gliding arc (GA) air plasma for enhancing ^137^Cs^+^ removal from aqueous EAFD suspensions. Plasma parameters were optimized based on their effects on water chemistry and reactive oxygen and nitrogen species formation. Under optimal conditions (200 W, 40 LPM, and 30 s), significant acidification and accumulation of nitrate and nitrite were observed, leading to matrix destabilization. The activity concentration decreased from 106.7 to 39.3 Bq g^−1^ (∼63.2% removal), while the dose rate was reduced by ∼78.3%. The results confirm effective radionuclide mobilization and demonstrate the potential of GA plasma for rapid EAFD remediation.

## Abstract 2

Gliding arc plasma generates reactive species that acidify water and destabilize the oxide matrix of electric arc furnace dust, enabling the rapid mobilization of ^137^Cs^+^.

## Graphical abstract

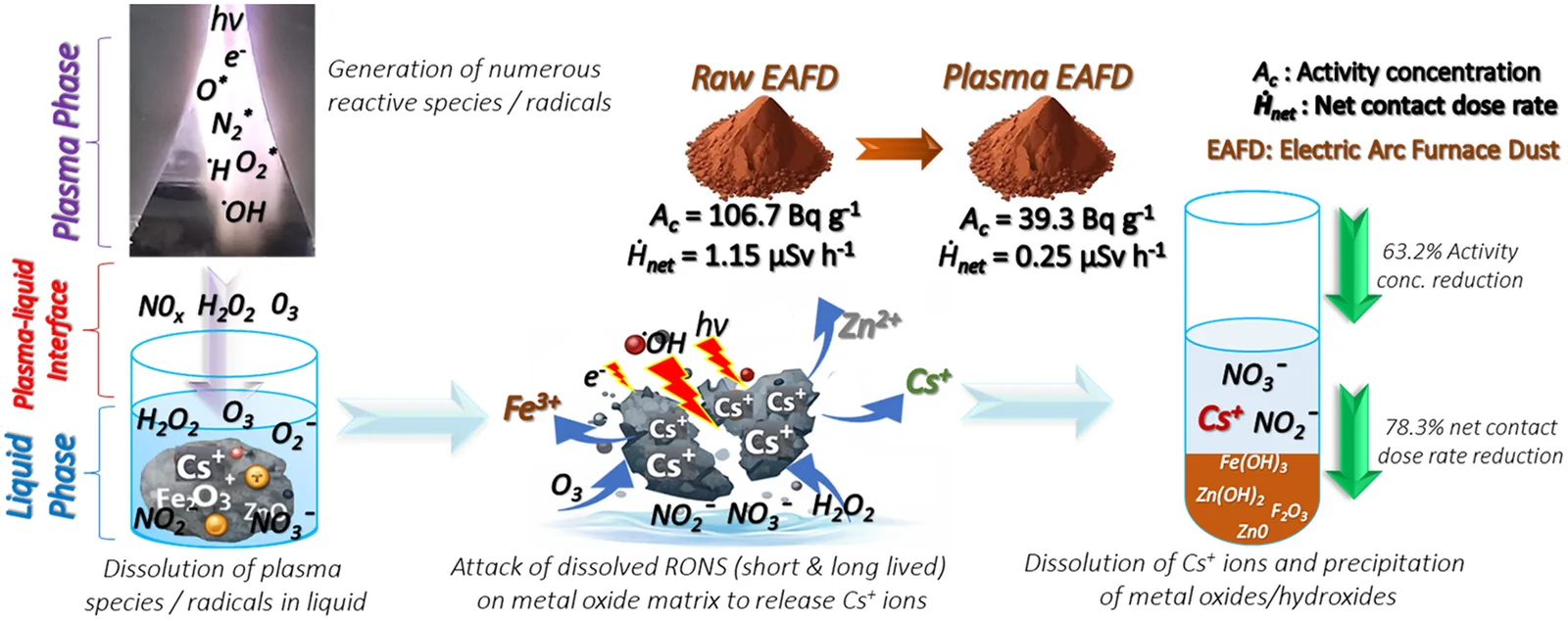

## 1. Introduction

Electric arc furnace dust (EAFD) is a hazardous by-product generated during steel production in electric arc furnaces.^1^ It contains high concentrations of heavy metals, such as iron, zinc, and lead, along with trace amounts of radionuclides that may originate from contaminated scrap materials.^2,3^ Among these radionuclides, cesium-137 (^137^Cs^+^) ions are of particular concern due to their mobility, environmental persistence, and contribution to radiological exposure.^4,5^ In EAFD, ^137^Cs^+^ ions are often trapped or associated within Fe- and Zn-based oxide matrices, making their removal challenging.^4,5^ The presence of such radionuclides increases the environmental and occupational risk of handling, recycling, or disposing of EAFD, highlighting the need for effective and sustainable remediation strategies.^4,5^

Conventional treatment approaches for radionuclide-contaminated waste typically rely on physicochemical separation, chemical extraction, and stabilization techniques to reduce environmental mobility and radiological risk.^4–7^ In the case of ^137^Cs-contaminated electric arc furnace dust (EAFD), several studies have applied solid–liquid extraction or lixiviation processes using acidic and aqueous media.^4–6^ Mineral acids, such as HNO_3_ and H_2_SO_4_, have demonstrated removal efficiencies exceeding 90%, while water-based systems achieved slightly lower but still significant decontamination levels.^4^ The efficiency of ^137^Cs^+^ removal has been shown to depend strongly on the solvent type, pH, solid-to-liquid ratio, and mineralogical composition of the dust.^4–7^ In addition to direct leaching, Sopapan *et al.*^5^ have proposed recovery-oriented strategies in which dissolved ^137^Cs is subsequently captured using cation-exchange resins, enabling potential reuse of the radionuclide. Complementary treatment of secondary liquid waste streams *via* co-precipitation has further enhanced the overall decontamination performance, often achieving greater than 99% removal from wastewater. Alternatively, Issarapanacheewin *et al.*^6^ investigated stabilization and immobilization approaches, including the incorporation of contaminated EAFD into cementitious matrices with activated carbon additives, to suppress radionuclide migration and improve long-term storage safety. Although these conventional methods have demonstrated high removal efficiencies, they often involve significant chemical consumption, secondary waste generation, or long-term stability concerns, thereby highlighting the need for optimized and sustainable treatment strategies. While these methods can be effective, they often require large chemical inputs, high energy consumption, or generate secondary waste streams. Moreover, the strong matrix binding of ^137^Cs^+^ within oxide structures limits the efficiency of simple washing or extraction techniques. Therefore, alternative treatment technologies that can destabilize the oxide matrix and enhance radionuclide mobilization under controlled conditions are highly desirable.

Non-thermal plasma has emerged as a promising advanced oxidation technology for environmental remediation.^8–11^ Unlike thermal plasma, where all species are at thermal equilibrium at high temperatures, non-thermal plasma operates under non-equilibrium conditions, where the electron temperature is significantly higher than the bulk gas temperature, enabling the generation of reactive species without uniformly heating the entire system.^9–12^ When applied in plasma–liquid systems, it generates a range of reactive oxygen and nitrogen species (RONS), including radicals, excited molecules, and oxidants, capable of inducing strong chemical transformations.^9,10,13^ These reactive species can modify water chemistry, promote acidification, and enhance oxidative reactions without the need for additional chemical reagents.^11,14^ Among various plasma configurations, gliding arc (GA) plasma offers advantages such as relatively simple design, atmospheric-pressure operation, and efficient generation of reactive species suitable for plasma–liquid interactions.^15–17^

Although non-thermal plasma has been widely studied for wastewater treatment,^9–11,17,18^ pollutant degradation,^9–11,19^ and microbial inactivation,^20,21^ its application in the treatment of radionuclide-bearing metallurgical waste remains limited. Recent studies have demonstrated the growing potential of plasma-based technologies in radioactive waste management.^22–24^ Nam *et al.*^22^ showed that underwater microwave plasma can be successfully applied for the decontamination of radioactive oxide films, such as chalk river unidentified deposits formed on nuclear power plant components. In that study, highly reactive plasma-generated species enabled efficient removal of deposited radionuclides, achieving up to 96.9% cobalt removal while minimizing the need for chemical additives and reducing secondary waste generation. Similarly, Kar *et al.*^24^ investigated the use of non-thermal microwave atmospheric pressure plasma jets for the decontamination of plutonium-based synthetic radioactive wastes, in which optimized plasma conditions—particularly oxygen-containing discharges—achieved removal efficiencies of approximately 92%. In addition, Zabulonov *et al.*^23^ coupled non-thermal plasma and sorption systems for the treatment of liquid radioactive wastes derived from the Chornobyl nuclear site, demonstrating greater than 90% removal of Cs-137 and Sr-90 with relatively low energy consumption and minimal solid residue generation. These studies collectively highlight the capability of plasma-generated reactive species to induce surface modification, oxidative degradation, and radionuclide mobilization under controlled conditions.

However, despite these advances, the application of plasma technology to radionuclide-bearing metallurgical wastes, such as electric arc furnace dust (EAFD), remains largely unexplored. In particular, the potential of gliding arc (GA) plasma to destabilize complex Fe- and Zn-based oxide matrices and enable controlled mobilization of trapped ^137^Cs^+^ has not yet been systematically examined. Given the strong association of radionuclides within oxide structures, understanding plasma-induced matrix modification is critical for developing effective remediation strategies.

The novelty of this work lies in demonstrating that GA plasma can function as a chemical activation platform, in which plasma-generated reactive species, localized acidification, and oxidative restructuring act synergistically to destabilize the oxide matrix and promote radionuclide release. This integrated plasma–chemical approach establishes a new pathway for controlled ^137^Cs^+^ mobilization and offers a promising strategy for plasma-assisted radiological risk reduction in industrial waste management.

In this study, gliding arc air plasma was applied to aqueous EAFD suspensions to investigate its effectiveness in removing trapped ^137^Cs^+^ and reducing radiological parameters. The electrical characteristics of the GA discharge were first analyzed to confirm stable plasma generation during plasma–liquid interactions. Subsequently, the physicochemical properties of water and the formation of reactive species were systematically examined under varying applied power, airflow rate, and treatment time to determine optimal operating conditions. The impact of plasma treatment on activity concentration and net contact dose rate was then evaluated, and morphological changes in EAFD particles were characterized using SEM. Based on these combined experimental findings, a comprehensive mechanism for plasma-assisted ^137^Cs^+^ removal was proposed.

## 2. Materials and methods

### 2.1 Materials

Raw electric arc furnace dust (EAFD) was collected from a steel manufacturing facility in Thailand. The main chemical compounds present in EAFD are oxides of zinc and iron (ZnO and Fe_2_O_3_);^25^ the detailed chemical composition of EAFD was reported by Yubonmhat *et al.*^25^ The material was air-dried and stored in sealed polyethylene containers prior to use. The initial activity concentration of raw EAFD was confirmed to be within permissible laboratory handling limits before experimentation. Tap water was used for preparing aqueous suspensions and for plasma–water characterization studies.

For plasma treatment experiments, 200 g of raw EAFD was dispersed in 2000 mL of tap water to prepare a homogeneous suspension. The mixture was stirred thoroughly to ensure uniform dispersion before plasma exposure.

### 2.2 Gliding arc (GA) plasma setup

The gliding arc (GA) plasma system was operated at atmospheric pressure using ambient air as the plasma-forming gas. The experimental configuration is shown schematically in Fig. 1(a). The GA reactor consisted of two identical leaf-shaped stainless-steel electrodes arranged in a diverging geometry. The arc was initiated at the narrow gap between the electrodes and was stretched upward along the electrode length by the airflow, producing a dynamic, high-current gliding arc discharge in contact with the liquid surface 5 cm from the leaf-shaped electrodes.

**Fig. 1:**
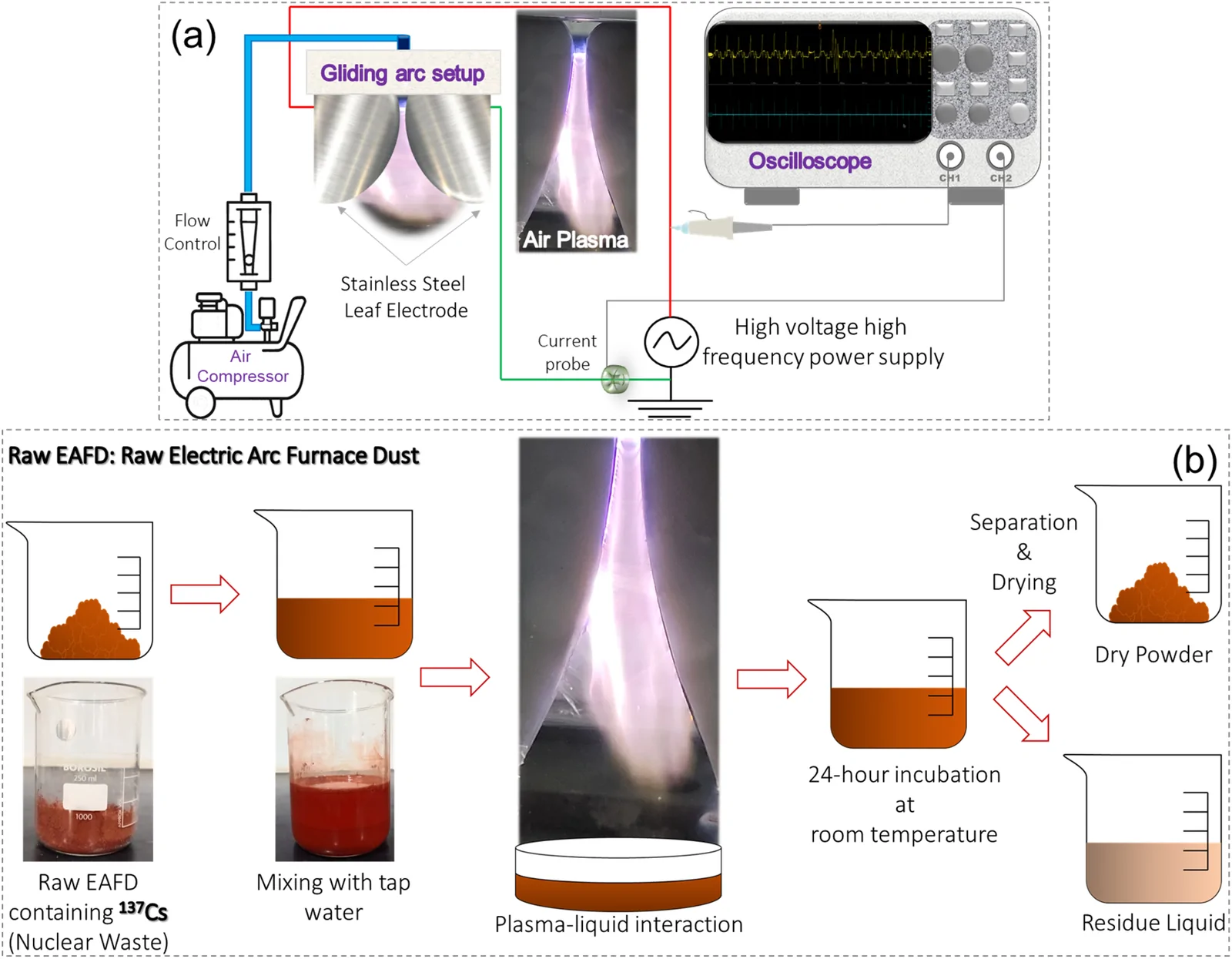
Experimental schematic of the GA plasma system and treatment process. (a) Schematic of the gliding arc plasma generation setup with corresponding voltage and current measurements. (b) Schematic of the GA plasma treatment of an aqueous EAFD suspension, followed by phase separation and drying to obtain the plasma-treated EAFD powder and residual liquid fraction.

Compressed air was supplied using an air compressor and regulated through a calibrated rotameter to control the flow rate. The experimental operating window (0–200 W applied power, 0–40 L min^−1^ air flow rate, and 0–30 s treatment time) was carefully defined to avoid excessive thermal effects and hydrodynamic disturbances during plasma–liquid interaction. Higher applied power or prolonged exposure could lead to heating of the suspension, potentially altering the physicochemical properties of the solution, accelerating evaporation, and influencing the stability of reactive species. Similarly, excessive airflow could cause splashing, sample loss, contamination of the surroundings with radioactive sample, and inconsistent plasma–liquid contact.

Based on the optimization study described in Section 3.3, the selected operating conditions were 200 W applied power, 40 L min^−1^ air flow rate, and 30 s treatment time, which ensured stable discharge generation, effective reactive species formation, and controlled treatment without thermal degradation of the sample. For each experiment, 20 mL of the prepared EAFD suspension was placed in a glass Petri dish and subjected to plasma treatment under these controlled conditions.

### 2.3 Electrical characterization

Electrical characterization of GA plasma was performed by recording voltage–current waveforms during plasma operation. The discharge voltage was measured using a 1000× high-voltage probe (Tektronix P6015A).^26,27^ The discharge current was monitored using a Rogowski coil current probe (CM-100-MG, Ion Physics Corporation) connected in series with the grounded electrode.

The voltage and current signals were recorded simultaneously using a digital oscilloscope (Tektronix MSO44 4-BW-500). These measurements were used to analyze discharge behavior and confirm stable plasma generation under optimized operating conditions.

### 2.4 Optical characterization

The generation of reactive species/radicals in the GA plasma system was investigated using optical emission spectroscopy (OES). Air was used as the working gas to produce non-thermal plasma under atmospheric conditions. The emission spectra of the plasma were recorded using an optical fiber coupled to a spectrometer (Aurora 4000).

For the GA configuration, the optical fiber probe was positioned at a distance of 10 cm from the plasma discharge zone, aligned along the direction of radical propagation. This placement enabled the detection of reactive species/radicals transported downstream from the discharge region. The collected spectra were used to identify active radicals and excited species generated during the plasma treatment of the EAFD suspension.

### 2.5 Plasma treatment procedure

For cesium-137 removal experiments, 20 mL aliquots of the aqueous EAFD suspension were treated with GA plasma under optimized conditions (200 W, 40 L min^−1^, 30 s) with experimental constraints. The operating parameters were within safe working limits and did not cause sample overheating, splashing, or any observable deterioration of the suspension. After treatment, the suspension was centrifuged to separate the solid and liquid phases.

The solid fraction (plasma-treated EAFD) was dried at 60 °C in an oven and then at room temperature and stored for further analysis. The supernatant liquid was also collected for activity measurement.

### 2.6 Measurement of physicochemical properties and reactive species

To evaluate plasma–liquid interactions and reactive species formation, tap water was treated under the same optimized plasma conditions. The changes in physicochemical properties, including pH, total hardness, total alkalinity, and total carbonates, were measured using commercially available water test strips (Inkbird 7-in-1).

Reactive oxygen and nitrogen species (RONS), including nitrate (NO_3_^−^), nitrite (NO_2_^−^), hydrogen peroxide (H_2_O_2_), and dissolved ozone (O_3_), were first screened using semi-quantitative test kits (QUANTOFIX and Hanna ozone kit). The minimum detection limits of the kits were approximately 10 mg L^−1^ for NO_3_^−^, 1 mg L^−1^ for NO_2_^−^, 0.5 mg L^−1^ for H_2_O_2_, and 0.01 mg L^−1^ for dissolved O_3_, according to the manufacturer's specifications.^27^

### 2.7 Activity concentration and net contact dose rate

The activity concentration (Bq g^−1^) of raw EAFD, plasma-treated EAFD, and the corresponding liquid fractions was determined using a high-purity germanium gamma spectrometer by following the process given by Yubonmhat *et al.*^25^ For determination of the ^137^Cs activity released into the liquid fraction, 10 g of the dried waste form was digested and diluted with tap water to a final volume of 1 L. The resulting liquid sample was transferred to a Marinelli beaker (Model 138-G) and analyzed using the HPGe gamma spectrometer. The measured activity of the liquid fraction was normalized to the initial mass of the waste form used for digestion and expressed as Bq g^−1^ of the original waste form. The detector system was calibrated using a certified reference material for radioactivity measurements in water (Model EG-ML, Eckert & Ziegler Isotope Products, USA), prepared in the same type of Marinelli beaker used for sample measurements.

The net contact dose rate (µSv h^−1^) was measured using a calibrated radiation survey meter by placing the detector in direct contact with the sample surface. All measurements were performed in triplicate, and the results are reported as mean values with standard deviation where applicable.

### 2.8 Surface morphology analysis

The surface morphology of raw and GA plasma-treated EAFD samples was examined using field emission scanning electron microscopy (FE-SEM). A Hitachi SU5000 FE-SEM instrument was used for imaging.

Prior to analysis, the dried powder samples were mounted on aluminum stubs using conductive carbon tape. Micrographs were recorded at different magnifications to evaluate changes in particle morphology, surface texture, agglomeration behavior, and structural modifications after plasma treatment. SEM analysis was performed at 10 kV accelerating voltage to correlate surface structural changes with plasma-induced physicochemical modifications and ^137^Cs^+^ removal performance.

## 3. Results and discussion

### 3.1 Electrical characterization of GA plasma during plasma–liquid interaction

Fig. 2(a) shows the voltage–current waveforms of GA plasma during interaction with the aqueous EAFD suspension. The discharge exhibits highly dynamic and non-linear behavior characterized by periodic voltage spikes accompanied by corresponding current fluctuations. The applied voltage reaches peak values of several kilovolts (*V*_pp|max_ – 18.6 kV), while the discharge current shows transient pulses in the ampere range (*I*_pp|max_ – 32.1 A), indicating repetitive ignition and extinction events typical of gliding arc discharges.^9,28^

**Fig. 2:**
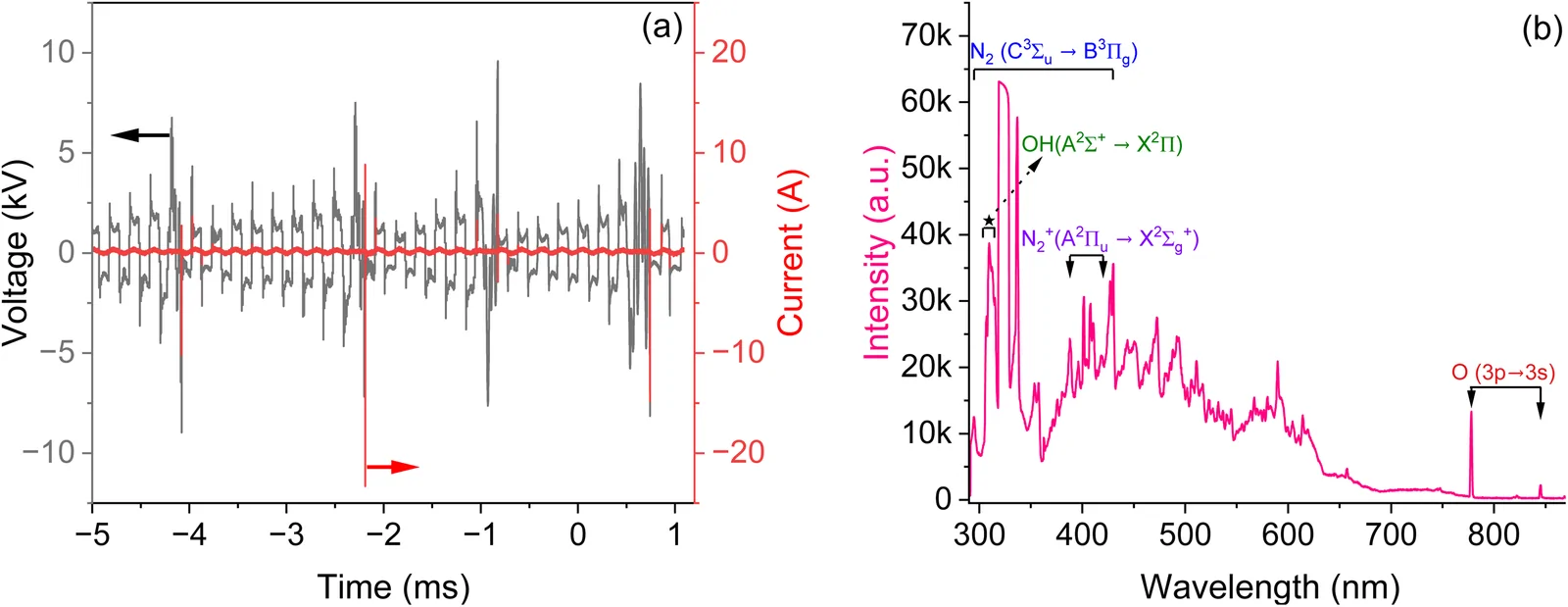
(a) Voltage–current waveforms and (b) optical emission spectra of GA air plasma during plasma–liquid interactions.

The waveform reveals a quasi-periodic regime with sharp voltage drops followed by rapid current surges, suggesting arc stretching, breakdown, and reattachment processes between the diverging electrodes. Such behavior is characteristic of a transitional discharge mode between thermal arc and non-thermal plasma regimes. The presence of intermittent high-current pulses indicates localized filamentary channels and enhanced energy deposition at the plasma–liquid interface.

These transient micro-discharges and current spikes enhance electron impact reactions, promoting the generation of gaseous reactive species/radicals, such as ˙OH, O, O_3_, and NO_*x*_, and solvated electrons at the plasma–liquid boundary.^29–33^ The strong electric field during voltage peaks facilitates ionization, excitation 
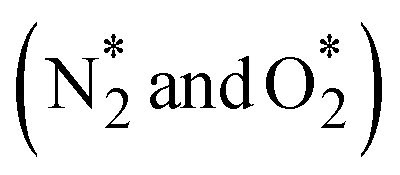
, and dissociation processes, while current pulses increase plasma density and reactive-species flux into the liquid phase.^26,34^ This dynamic discharge behavior is proposed to be directly responsible for the effective oxidative attack and dissociation of the EAFD metal oxide matrix, leading to the release of Cs^+^ into the aqueous medium.

### 3.2 Optical emission characterization of GA plasma during plasma–liquid interaction

The emission spectrum of air plasma generated by the GA system provides important insights into the reactive species responsible for ^137^Cs^+^ mobilization and radiological risk reduction in EAFD. As shown in Fig. 2(b), GA plasma exhibits several characteristic emission features associated with reactive species/radicals.

A prominent emission band corresponding to OH (A^2^∑^+^ → X^2^Π) transitions in the range of 306–310 nm was clearly observed, indicating the abundant formation of hydroxyl radicals (˙OH). These radicals possess extremely high oxidation potential and play a crucial role in initiating non-selective reactions at the solid–liquid interface. In the present system, ˙OH radicals contribute to the breakdown of surface bonds within Fe- and Zn-based oxide matrices (*e.g.*, Fe_2_O_3_ and ZnO), facilitating the release of entrapped Cs^+^ ions into the aqueous phase.

In addition, strong emissions from the N_2_ second positive system (C^2^∑_u_ → B^2^Π_g_) in the range of 290–350 nm were detected, confirming the presence of excited nitrogen species. These species are important precursors for the formation of reactive nitrogen species such as NO_*x*_, which subsequently dissolve in water to form nitrate (NO_3_^−^) and nitrite (NO_2_^−^) ions. The accumulation of these species contributes significantly to plasma-induced acidification, as observed experimentally, thereby enhancing matrix destabilization and promoting Cs^+^ leaching.

Furthermore, the presence of atomic oxygen emission lines in the range of 770–850 nm (3p → 3s) indicates the generation of reactive oxygen atoms (O˙). These species can participate in secondary reactions leading to the formation of longer-lived oxidants such as ozone (O_3_) and hydrogen peroxide (H_2_O_2_). The combined action of short-lived radicals (˙OH, O˙) and longer-lived oxidants creates a highly oxidative environment that accelerates the dissolution and structural weakening of the EAFD matrix.

Overall, the emission spectrum of GA plasma confirms the simultaneous generation of highly reactive ˙OH radicals, atomic oxygen, and nitrogen-derived species. This rich oxidative chemistry plays a key role in modifying the aqueous environment, destabilizing the oxide matrix, and enhancing the mobilization of ^137^Cs^+^. The strong presence of these reactive species highlights the effectiveness of the GA plasma system as a rapid and energy-efficient approach for radiological risk reduction in contaminated industrial waste.

### 3.3 Physicochemical properties and RONS generation in tap water after GA plasma treatment

Fig. 3 shows the changes in physicochemical parameters (pH, total hardness, total alkalinity, and carbonates) and RONS concentrations (NO_3_^−^, NO_2_^−^, H_2_O_2_, and diss. O_3_) in tap water after GA plasma treatment (measured after 24 hours of plasma treatment) under varying applied power, air flow rate, and treatment time. Optimization was performed sequentially by first varying the applied power (150–200 W), followed by air flow rate (20–40 L min^−1^), and finally treatment time (10–30 s) within experimental constraints.

**Fig. 3:**
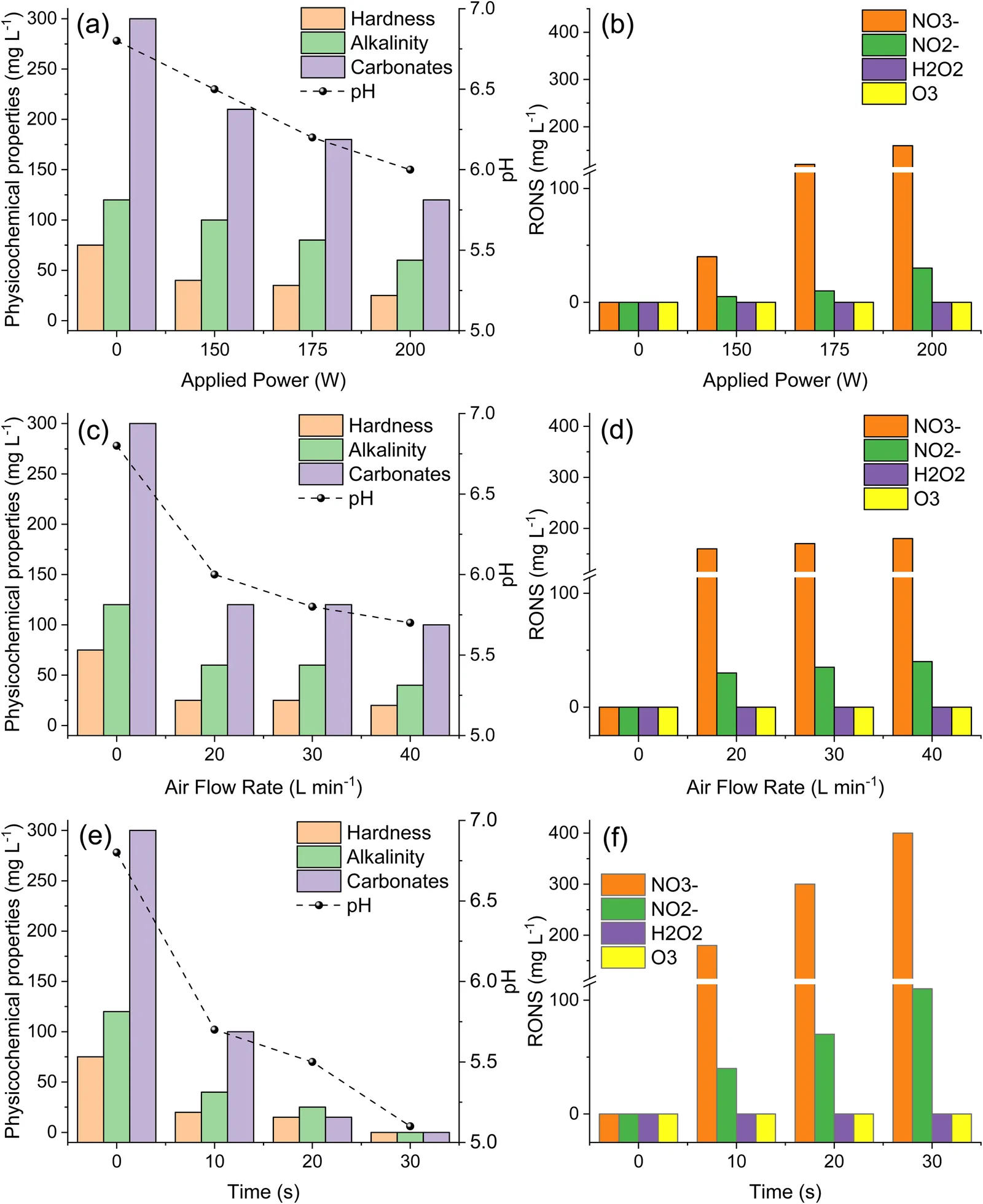
Changes in the physicochemical properties (pH, total hardness, total alkalinity, and total carbonates) and RONS concentrations (NO_3_^−^ ions, NO_2_^−^ ions, H_2_O_2_ and diss. O_3_) of tap water after GA plasma (measured after 24 h) treatment under varying (a and b) applied power, (c and d) air flow rates, and (e and f) treatment times, respectively.

#### 3.3.1 Effect of applied power

Increasing the applied power from 0 to 200 W resulted in a progressive decrease in pH from 6.8 to 6.0 with 10 s of treatment time and 20 L min^−1^ of air flow rate. Simultaneously, NO_3_^−^ concentration increased significantly from 0 to 160 mg L^−1^, while NO_2_^−^ increased from 0 to 30 mg L^−1^. No measurable H_2_O_2_ was detected under the tested conditions. Total hardness, alkalinity, and carbonate concentrations decreased markedly with increasing power.

The decrease in pH is attributed to enhanced plasma-induced formation of nitrogen oxides (NO_*x*_) in air, which dissolve into water to form nitric (HNO_3_, NO_3_^−^ ions) and nitrous (HNO_2_, NO_2_^−^ ions) acids.^30,32,34,35^ Higher applied power increases electron density and discharge energy, promoting excitation and dissociation of N_2_ and O_2_, thereby accelerating RONS generation.^27,36^ The concomitant reduction in alkalinity and carbonates indicates acid neutralization reactions,^37^ consuming bicarbonate and carbonate buffering species as follows:H^+^ + CO_3_^2−^ → HCO_3_^−^,H^+^ + HCO_3_^−^ → CO_2_ + H_2_O.

The reduction in total hardness suggests partial dissolution or transformation of Ca^2+^/Mg^2+^ buffering species under acidic conditions.

Among the tested powers, 200 W produced the highest RONS concentration and strongest acidification within experimental constraints and was selected as the optimum operating power for subsequent studies. This condition is particularly relevant for Cs^+^ removal, as acidic and oxidizing environments promote dissolution of the metal oxide matrix (Fe_2_O_3_, ZnO), trapping Cs^+^.^5,6,25^

#### 3.3.2 Effect of air flow rate

At the optimized power (200 W) within experimental constraints, increasing the air flow rate from 20 to 40 LPM further enhanced RONS generation. The NO_3_^−^ concentration increased from 160 to 180 mg L^−1^, while NO_2_^−^ rose from 30 to 40 mg L^−1^, accompanied by a decrease in pH from 6.0 to 5.7. A corresponding reduction in total hardness, alkalinity, and carbonate concentrations was also observed, indicating continued acidification and buffering capacity depletion. However, the magnitude of these changes was comparatively less pronounced than those induced by variations in applied power (Section 3.3.1) and treatment time (Section 3.3.3), suggesting that air flow rate plays a secondary role in governing the physicochemical evolution and RONS production under the investigated conditions.

Higher air flow improves the mass transfer of reactive nitrogen and oxygen species into the plasma zone and enhances plasma–liquid interfacial interaction. The increased availability of N_2_ and O_2_ promotes the formation of excited species 
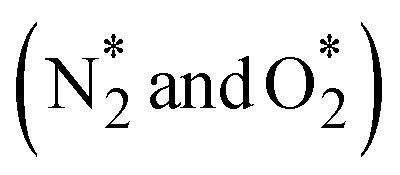
 and atomic radicals (O˙ and N˙), leading to higher NO_*x*_ production and subsequent aqueous acidification.^9,10,14,17,20^

The flow rate of 40 LPM provided maximum RONS generation and the strongest buffering capacity depletion and was therefore selected as the optimal airflow condition within experimental constraints. These conditions may favor matrix destabilization in EAFD by promoting acid-driven dissolution and oxidative attack.

#### 3.3.3 Effect of treatment time

Under optimized power (200 W) and flow rate (40 L min^−1^) within experimental constraints, increasing the treatment time from 10 to 30 s produced substantial cumulative effects. The pH decreased progressively from 5.7 to 5.1. NO_3_^−^ increased sharply from 180 to 400 mg L^−1^, while NO_2_^−^ increased from 40 to 110 mg L^−1^. Simultaneously, total hardness, alkalinity, and carbonates were completely depleted after 30 s of treatment.

The continuous accumulation of nitrate and nitrite indicates sustained NO_*x*_ dissolution and ongoing acid formation. The complete removal of carbonate buffering capacity at 30 s confirms strong acidification of the solution,^38^ generating conditions highly favorable for metal oxide dissolution as follows:ZnO + 2H^+^ → Zn^2+^ + H_2_O,Fe_2_O_3_ + 6H^+^ → 2Fe^3+^ + 3H_2_O.

Such acidic conditions are essential for destabilizing the EAFD matrix and liberating trapped Cs^+^ into the aqueous phase.^4,5,25^ Therefore, 30 s was selected as the optimum treatment time for subsequent plasma-assisted Cs^+^ removal experiments.

Although no detectable concentrations of H_2_O_2_ and dissolved O_3_ were observed after 24 h of GA plasma treatment, their absence in the analytical results does not imply negligible formation.^26,27,39^ Under plasma–liquid interactions, both H_2_O_2_ and O_3_ are transiently generated as primary reactive oxygen species such as ˙OH and O. However, in nitrogen-containing systems such as air plasma, these species readily react with nitrite (NO_2_^−^), leading to the formation of the more thermodynamically stable nitrate (NO_3_^−^).^26,39,40^ For instance, H_2_O_2_ can oxidize NO_2_^−^ to NO_3_^−^, while O_3_ reacts rapidly with NO_2_^−^ in aqueous media, promoting further NO_3_^−^ formation.^27,39^ Consequently, the rapid consumption of H_2_O_2_ and O_3_ explains their non-detectable residual concentrations and the simultaneous higher accumulation of NO_3_^−^ observed in the treated water.^26,27,39^

Despite their transient nature, ˙OH, H_2_O_2_, ˙O, O_3_, and other plasma-generated ROS play a crucial mechanistic role during plasma–liquid interactions.^9,10,32,33^ As strong oxidizing agents, ROS contribute significantly to the oxidative destabilization of the Fe_2_O_3_ and ZnO matrix, enhancing metal oxide dissolution and facilitating the release of trapped Cs^+^ into the aqueous phase.^4,5,25^ Therefore, even though they were not detected in the final solution, their *in situ* generation and rapid reaction kinetics are essential for initiating and sustaining the chemical pathways responsible for effective Cs^+^ removal.

### 3.4 Reduction of activity and net contact dose rate of EAFD after GA plasma treatment

Fig. 4 shows the activity concentration (*A*_c_) and net contact dose rate (*Ḣ*_net_) of raw and GA plasma-treated EAFD, together with the calculated removal efficiencies. The corresponding numerical values for solid and liquid fractions are summarized in Table 1.

**Fig. 4:**
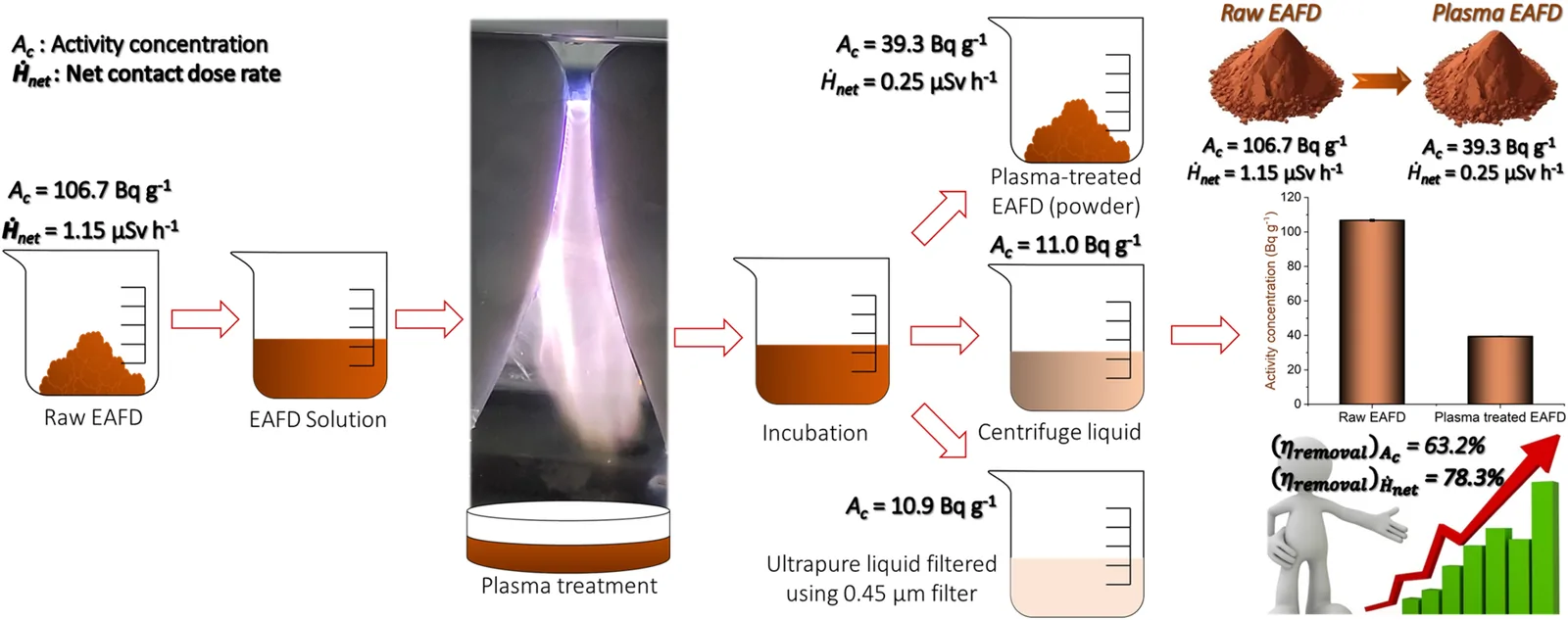
Activity concentration (*A*_c_) and net contact dose rate (*Ḣ*_net_) of raw and GA plasma-treated EAFD, along with the corresponding removal efficiencies.

**Table 1:** Activity concentration and net contact dose rate of raw and plasma-treated EAFD and the corresponding liquid fractions

Sample	Activity concentration (Bq g^−1^)	Net contact dose rate (µSv h^−1^)
Raw EAFD	106.745 ± 0.435^a^	1.15
Plasma-treated EAFD	39.338 ± 0.05^b^	0.25
Centrifuge supernatant solution	11.002 ± 0.06^c^	—
Ultrapure supernatant solution	10.871 ± 0.072^c^	—

The raw EAFD exhibited an *A*_c_ of 106.75 Bq g^−1^ with a net contact dose rate of 1.15 µSv h^−1^. Following GA plasma treatment under optimized conditions, the activity concentration significantly decreased to 39.34 Bq g^−1^, representing a removal efficiency of ∼63.2%. Simultaneously, *Ḣ*_net_ declined markedly to 0.25 µSv h^−1^, corresponding to a reduction of ∼78.3% relative to the untreated sample.

The larger reduction in net contact dose rate compared to activity concentration suggests not only a decrease in total radionuclide content but also a redistribution of radioactive species within the matrix. Plasma-induced acidification and oxidative reactions destabilize the Fe_2_O_3_ and ZnO matrix, promoting the release of trapped Cs^+^ from the particle surface and near-surface regions. The removal of surface-associated radioactivity contributes substantially to the observed decrease in contact dose rate.

Analysis of the liquid fractions further supports the proposed leaching mechanism. The centrifuged supernatant exhibited an activity concentration of 11.0 Bq g^−1^, while the ultrapure supernatant showed a similar value of 10.87 Bq g^−1^, with no statistically significant difference between them (*p* > 0.05). These results confirm the transfer of a fraction of the radionuclide inventory from the solid EAFD matrix into the aqueous phase during plasma treatment.

Each experiment treated 20 mL of the suspension for only 30 s. In total, 2 L of the suspension containing 200 g of EAFD was processed sequentially in 20 mL batches. Despite the very short exposure time, substantial reductions in both activity concentration and dose rate were achieved, with a relatively low specific energy input (≈0.084 kWh per L per 30 s treatment).

Compared with previous studies, the present approach operates with much shorter treatment times. Nam *et al.*^22^ reported 96.9% cobalt removal using underwater Ar + H_2_ microwave plasma, but the treatment required 120 min of exposure. Zabulonov *et al.*^23^ achieved >90% removal of Cs-137 and Sr-90 from liquid radioactive waste using a coupled plasma-sorption system, with processing times of 15–45 min and an energy consumption of about 10 kW h for 15–20 L h^−1^ treatment. Chemical extraction methods, such as those reported by Akharawutchayanon *et al.*^4^ and Sopapan *et al.*,^5^ achieved high removal efficiencies but required strong acids or additional chemical precipitation steps.

In contrast, the GA plasma treatment investigated in the present study resulted in significant ^137^Cs activity reduction within only 30 s of treatment per batch and without direct addition of strong acids. This rapid response is likely associated with the combined effects of plasma-induced acidification and plasma-generated reactive species at the plasma–liquid interface, which can promote destabilization and dissolution of radionuclide-associated matrix components. However, because a complete phase-wise ^137^Cs activity balance and detailed energy assessment were not performed in the present study, the process cannot yet be considered quantitatively superior in terms of energy efficiency or absolute radionuclide removal. Nevertheless, the short treatment time and avoidance of strong chemical additives highlight the potential of GA plasma treatment as a rapid approach for reducing ^137^Cs activity in EAFD-derived waste forms and warrant further investigations at a larger scale.

### 3.5 Surface morphology of raw and plasma-treated EAFD

SEM micrographs of raw EAFD (Fig. 5(a)) and GA plasma-treated EAFD (Fig. 5(b)) at different magnifications are shown in Fig. 5. The untreated EAFD exhibits highly agglomerated particles with irregular morphology and heterogeneous size distribution. At higher magnification, the raw sample consists of a mixture of spherical and angular particles with relatively rough and compact surfaces. Fine particulates are attached to larger particles, forming dense aggregates, which suggests strong interparticle adhesion and a compact oxide matrix structure.

**Fig. 5:**
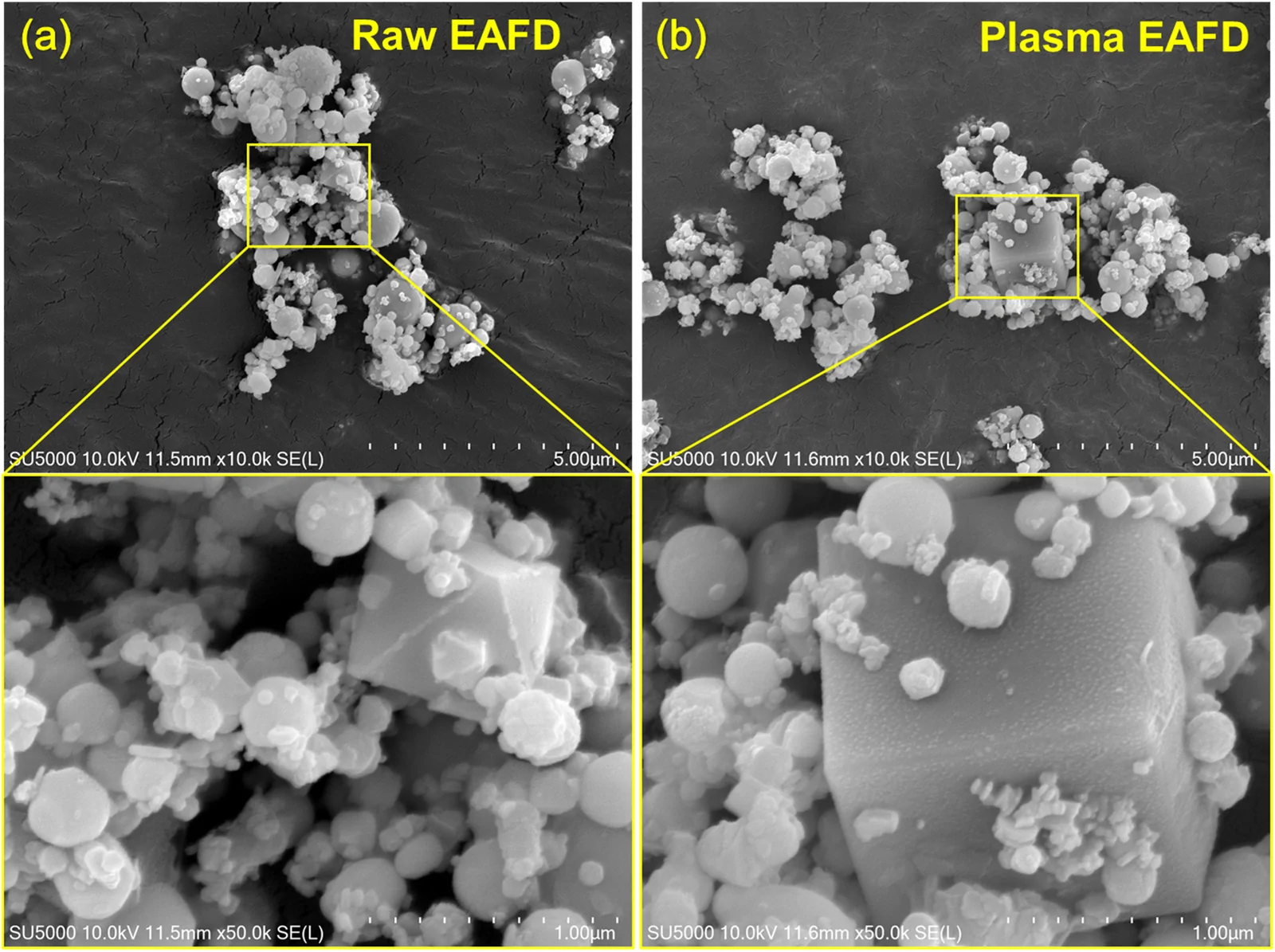
SEM micrographs showing the surface morphologies of (a) raw EAFD and (b) plasma-treated EAFD.

In contrast, the plasma-treated EAFD shows noticeable morphological modification. Although spherical particles remain present, the surface appears comparatively smoother and more defined in shape. The larger particles exhibit clearer boundaries and reduced fine-particle agglomeration. At higher magnification, the plasma-treated sample displays partial surface restructuring, including localized smoothing and slight surface texturing, indicating plasma-induced thermal and chemical effects.

The observed morphological changes can be attributed to the combined action of plasma-generated reactive species and localized thermal micro-discharges during GA plasma treatment. The oxidative and acidic environment promotes partial dissolution of surface-associated phases (*e.g.*, Fe_2_O_3_ and ZnO), weakening interparticle binding and disrupting the compact matrix. This structural modification likely facilitates the release of trapped Cs^+^ by increasing surface accessibility and promoting matrix destabilization.

Hence, SEM analysis supports that GA plasma treatment induces measurable microstructural alterations in EAFD, supporting the proposed mechanism of plasma-assisted matrix degradation and radionuclide mobilization.

### 3.6 Proposed mechanism of Cs^+^ removal during GA plasma treatment

The overall proposed pathway responsible for Cs^+^ removal from EAFD during GA plasma treatment is shown in Fig. 6, integrating the physicochemical evolution of the aqueous phase, morphological transformation of the solid matrix, and the radiological reduction results discussed previously.

**Fig. 6:**
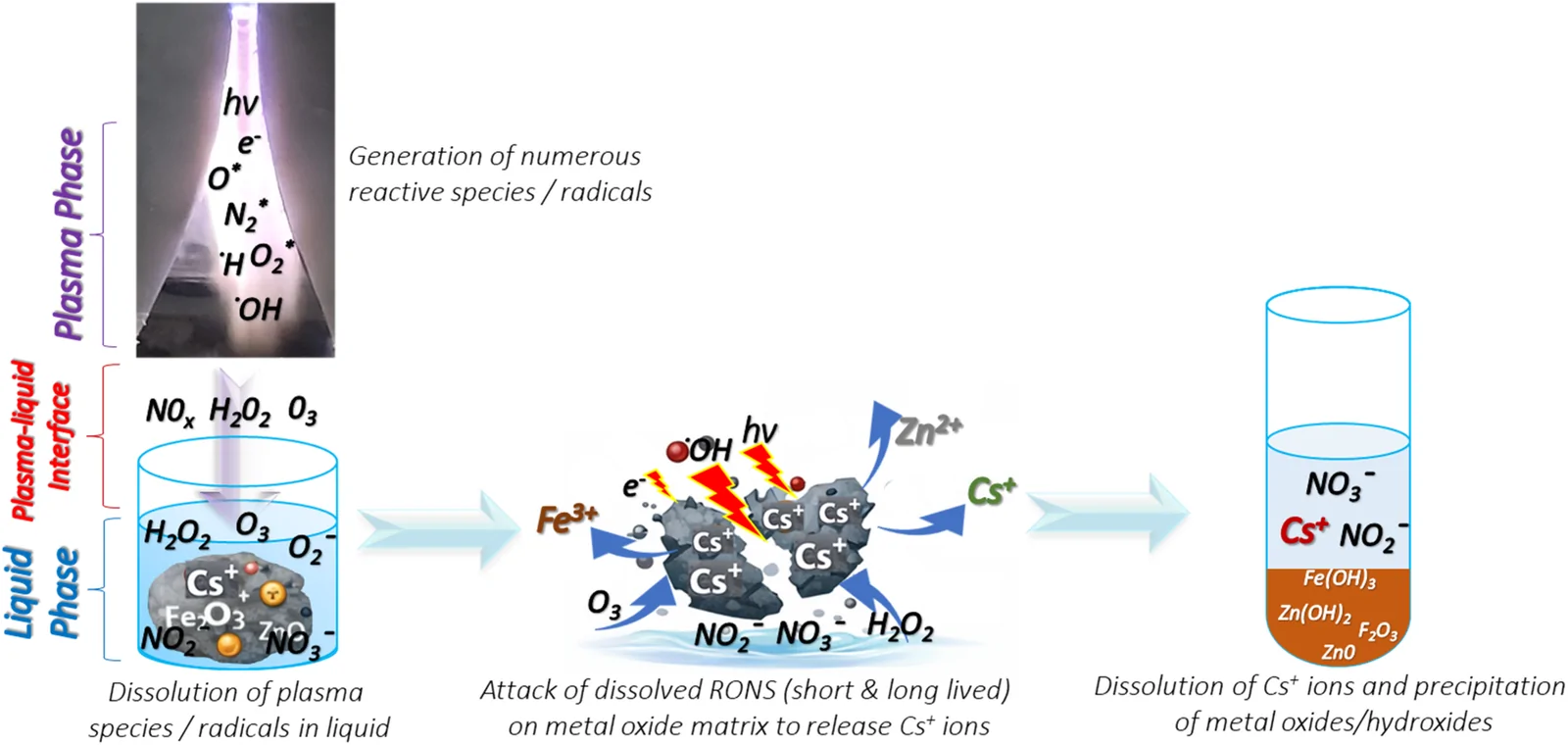
Proposed reaction mechanism for Cs^+^ removal during the GA plasma treatment of an aqueous EAFD suspension.

The gliding arc discharge generates a chemically active plasma zone rich in energetic electrons and reactive species/radicals such as ˙OH, ˙O, O_3_, *etc.*^9,14,17,27,32^ Upon interaction with the aqueous suspension, these species create an increasingly acidic and oxidative environment, as evidenced by the progressive decrease in pH, alkalinity, hardness, and carbonates, together with substantial accumulation of nitrate and nitrite ions under optimized operating conditions.^16,26,35,40^

The sustained modification of water chemistry destabilizes the Fe- and Zn-based oxide matrix that hosts Cs^+^ within the EAFD structure.^4,6,25^ SEM observations confirm that plasma treatment alters particle surface morphology, indicating matrix weakening and structural reorganization. This physicochemical disruption facilitates the release of previously immobilized Cs^+^ into the liquid phase.^5,7,25^

The measurable activity detected in the supernatant fractions, together with the significant reduction in activity concentration and net contact dose rate of the treated solid, confirms that radionuclide transfer from the solid matrix to the aqueous phase occurs effectively under optimized plasma conditions within experimental constraints. The larger relative decrease in the dose rate compared to bulk activity further suggests preferential removal of surface-associated radionuclides, which are the most influential in contact radiation exposure.

In summary, GA plasma treatment operates through a synergistic mechanism combining plasma-induced chemical activation,^9,15,16,34^ matrix destabilization, and radionuclide mobilization.^22–24^ The process converts electrical discharge energy into chemical driving forces that promote Cs^+^ liberation from the trapped waste matrix, ultimately reducing the radiological hazard of EAFD.

### 3.7 Scale-up challenges and economic considerations

Although the present study demonstrates the potential of GA plasma treatment for reducing ^137^Cs activity in EAFD-derived waste forms, translation of the process from laboratory scale to industrial application requires consideration of several technical and economic factors. At larger scales, maintaining uniform plasma generation and treatment conditions throughout a larger quantity of heterogeneous EAFD may be challenging. Reactor configuration, electrode design, plasma power distribution, treatment residence time, and process throughput would therefore require optimization for continuous or semi-continuous operation. In addition, electrode durability and the stability of plasma operation in the presence of variable EAFD composition should be evaluated during long-term operation.

The treatment of radioactive EAFD also requires appropriate radiation-safety measures. Industrial implementation would require controlled handling and containment of the radioactive feed material, suitable shielding of the treatment system, remote or automated operation where necessary, and appropriate management of the treated solid and liquid fractions. These requirements may increase the capital and operating costs compared with conventional non-radioactive plasma applications.

From an economic perspective, the major operating costs are expected to be associated with electricity consumption for plasma generation, reactor and electrode maintenance, radiation protection, and downstream management of the treated fractions. Overall economic performance will therefore depend strongly on the treatment capacity, plasma energy efficiency, reactor configuration, and degree of ^137^Cs removal achieved. Conversely, the potential advantages of the GA plasma process include rapid treatment, reduced dependence on conventional chemical reagents, and the possibility of integrating plasma treatment with existing radioactive-waste management processes.

At the present laboratory scale, sufficient data are not available to establish a reliable techno-economic assessment or to claim economic superiority over established radioactive-waste treatment technologies. A comprehensive techno-economic analysis should therefore be conducted at pilot scale by considering capital expenditure, electricity and material consumption, treatment throughput, maintenance, radiation-safety infrastructure, and downstream waste-management costs. Such assessment will be important for determining the economic feasibility and optimal operating conditions of GA plasma treatment for industrial-scale radioactive EAFD management.

## 4. Conclusion

This study demonstrates that GA air plasma treatment is an effective approach for enhancing ^137^Cs^+^ removal from aqueous EAFD suspensions and reducing the associated radiological hazard. Plasma–liquid interactions generated reactive oxygen and nitrogen species that significantly modified the physicochemical properties of water, leading to acidification and depletion of buffering components. These chemically active conditions destabilized the Fe- and Zn-based oxide matrix of EAFD, facilitating the release and transfer of trapped ^137^Cs^+^ into the aqueous phase. Morphological analysis further confirmed structural alterations of dust particles after treatment. Overall, GA plasma treatment provides a promising, environmentally friendly strategy for the remediation and radiological risk reduction of radionuclide-bearing metallurgical waste.

## Author contributions

Vikas Rathore: conceptualization, data curation, formal analysis, investigation, validation, visualization, writing – original draft. Arlee Tamman: methodology, resources, writing – review and editing. Klitsadee Yubonmhat: methodology, resources, writing – review and editing. Kewalee Nilgumhang: formal analysis, writing – review and editing. Dechanun Choomjun: investigation, data curation, writing – review and editing. Shaima Masae: investigation, data curation, writing – review and editing. Pattachai Bor-in: investigation, data curation, writing – review and editing. Grittima Kongprawes: writing – review and editing. Tejal Barkhade: writing – review and editing. Arunsinh B. Zala: writing – review and editing. Subash C. B. Gopinath: writing – review and editing. Tijjani Adam: writing – review and editing. Siti Khadijah Zaaba: writing – review and editing. Falguni Bhabhor: writing – review and editing. Sujoy Chattaraj: writing – review and editing. Mudtorlep Nisoa: project administration, supervision, writing – review and editing.

## Conflicts of interest

No potential conflicts of interest were reported by the authors.

## Data Availability

All data that support the findings of this study are included within the article.
